# Cuando el aire se hizo de dominio público

**DOI:** 10.7705/biomedica.7552

**Published:** 2024-05-30

**Authors:** Luis Jorge Hernández-Flórez

**Affiliations:** 1 Profesor asociado, Facultad de Medicina, Universidad de los Andes, Bogotá, D. C., Colombia Universidad de los Andes Universidad de los Andes Bogotá D. C. Bogotá

El aire que respiramos es parte de la historia de la Medicina y de la relación entre el ambiente y la salud. Ya Hipócrates, en el siglo V antes de Cristo y en su obra *Aires, Aguas y Lugares*, menciona que “El curso del sol, de la luna y de los astros ocurre a causa del viento. Las afecciones surgen cuando el aire es abundante o escaso, es compacto o lleno de impurezas”. La enfermedad en la medicina clásica grecorromana resultaba de un desequilibrio entre una armonía interna del individuo con la naturaleza representada en cada uno de los cuatro elementos definidos por Empédocles: aire, tierra, agua y fuego.

Por más de dos mil años se pensó que el paludismo, o malaria, era un mal que provenía de los pantanos, como lo demuestra la etimología de ambos nombres. Paludismo viene de la palabra “palude”, que en italiano significa “pantano”, mientras que malaria hace referencia al “mal aire” que emana de los pantanos y que suponían era el causante de la enfermedad ^1^.

El aire era un constructo del proceso salud-enfermedad y marcó el inicio de la Modernidad con la teoría miasmática, en la cual se sostenía que había algo en el aire que favorecía la aparición de la enfermedad. Fue la epidemia de peste la que puso en crisis el pensamiento mágico religioso de la edad media y, entonces, el aire hizo su entrada como categoría que servía para explicar, por primera vez en mil años, fenómenos dentro de la misma naturaleza, sin recurrir a fuerzas sobrenaturales. El miasma era un aire contaminado por “materias corruptas o aguas estancadas” y que producía enfermedades.

El miasma era el aire que contenía algo desconocido de la misma naturaleza y que penetraba por nuestra respiración, por la boca y por la piel, por lo cual era clave establecer barreras de contacto entre ese aire impuro y la persona, mediante perfumes, vestidos, pelucas, mascaras, túnicas. Esta teoría miasmática prevaleció en los siglos XVI y XVII; sin embargo, perdura como paradigma explicativo hasta nuestros días, por ejemplo, cuando hablamos de los “fríos” que “penetran” el organismo, lo cual se refleja en las creencias populares «para prevenir o tratar los problemas respiratorios el niño debe usar una camiseta roja de tela de bayetilla, la cual no permite que ‘le entre frío’ y empeore; también, se usa para prevenir las enfermedades respiratorias a repetición» ^2^.

Una vez dejada atrás la teoría del miasma, al menos como paradigma y no como creencia, surgió en el siglo XIX la teoría bacteriológica y lo que se conoce como la primera revolución epidemiológica del agente, huésped y medioambiente. Entonces, el aire vuelve a tener un papel relevante en los conceptos de prevención y sanidad que surgieron por la revolución industrial en Inglaterra y Estados Unidos, mediante la cual el entorno, el agua, el saneamiento y la ventilación, se hicieron importantes para preservar la vida y la salud de la nueva generación de obreros. Aparece Edwin Chadwick del movimiento británico de salud pública: “Ya en 1832 una mortífera epidemia de cólera había traído al primer plano los problemas de salud pública y la necesidad de buscar soluciones, si bien el reto de la alta mortalidad de las ciudades industriales -enfrentadas al aire contaminado, el hacinamiento, el vertido incontrolado de residuos, el amontonamiento de basuras o los problemas de garantía de abastecimiento de agua potable- venía planteándose desde tiempo atrás” ^3^. El aire entró en la categoría de factor de riesgo y hace parte de la triada epidemiológica de agente, huésped y medioambiente hasta nuestros días.

Sin embargo, fue en la llamada segunda revolución epidemiológica, término acuñado por Milton Terris en 1994 para designar la epidemiología de las enfermedades crónicas no transmisibles después de la Segunda Guerra Mundial, en la que la calidad del aire se consideró más rigurosamente como un factor de riesgo para eventos mórbidos y mortales, como el cáncer de pulmón y la enfermedad pulmonar obstructiva crónica. Ya desde finales del siglo XIX, se incrementaron y documentaron los casos graves de contaminación del aire, como el episodio de niebla que cubrió Londres en 1873 y causó 268 muertes por bronquitis, fenómeno que se repitió en la misma ciudad en el año 1952, con una duración de tres días y más de 4.000 muertes. En 1956, el parlamento inglés promulgó la llamada «Ley del aire puro».

La calidad del aire se ha considerado como un factor de riesgo desde el modelo canadiense de salud-enfermedad del año 1974 y como factor determinante social en el modelo de la Organización Mundial de la Salud (OMS) en el año 2005. Desde el 2022, el Consejo de Derechos Humanos de las Naciones Unidas declaró el acceso a un “medio ambiente limpio, saludable y sostenible” como un derecho humano universal y se considera la contaminación del aire como un problema público, “que no solamente concierne a los responsables de la contaminación sino también a quienes podrían sufrir las consecuencias” ^4^. Incluso países como la China consideran la contaminación del aire como un problema de seguridad nacional ^5^.

En el “Modelo de determinantes sociales y ambientes” de la OMS, la calidad del aire hace parte del factor determinante estructural, ya que está dado por el modelo de desarrollo, producción y consumo territorial, así como por las políticas de adaptación y mitigación al cambio climático. También, hace parte del factor determinante intermedio por ser parte de los ambientes construidos, tales como los hogares, las escuelas y, en general, a los ámbitos de la vida cotidiana.

Se distinguen, al menos, tres grados de contaminación del aire: uno macro, que es medido por las redes de monitoreo de calidad del aire y en el que se cuantifica la inmisión de los llamados contaminantes-criterio; uno intermedio o meso, que se mide en los espacios intramurales ya señalados de los ámbitos de vida cotidiana; y un tercer grado, o exposoma, que es la nube de exposición de cada persona a los contaminantes del aire. Es importante, aquí, distinguir las mediciones ambientales de las sanitarias y la necesidad de trabajo interdisciplinario para abordar las mediciones de calidad del aire, acercando cada vez más la medición a lo que respira cada persona, inclusive, avanzando en la medición de biomarcadores de exposición, susceptibilidad y daño.

Hoy, también, se considera relevante la gobernanza del aire que implica monitorear desde la ciudadanía el cumplimiento de los planes de descontaminación del aire a nivel territorial y analizar las responsabilidades.

En Colombia, la cobertura de la red de monitoreo del aire sigue siendo poca, menor del 25 %; a partir de esta información, se sabe que, a nivel nacional, las zonas que mayor afectación presentan por importantes grados de contaminación atmosférica son: el área metropolitana del valle de Aburrá (Antioquia), las localidades de Puente Aranda, Carvajal y Kennedy en Bogotá, el municipio de Ráquira en Boyacá y la zona industrial de ACOPI en el municipio de Yumbo en el Valle del Cauca.

En el presente número de *Biomédica*, aparece un artículo titulado “Calidad del aire y su impacto en la salud pública”; es un insumo importante que aporta información para orientar una mejor toma de decisiones a nivel nacional y territorial, para evaluar y reformular las políticas públicas de calidad de aire y salud.

Para mejorar la toma de decisiones en el ámbito de la gestión de la calidad del aire, es esencial ampliar y modernizar la red de monitoreo del aire y promover el acceso a datos precisos y actualizados. Las políticas deben basarse en pruebas científicas y evaluaciones de impacto, con una normativa estricta sobre emisiones y el fomento de las energías limpias y el transporte sostenible.

Es clave hacer adecuada comunicación social del riesgo, formación de ciudadanía y posicionar en la agenda pública el tema de calidad del aire y la salud, así como avanzar en una gobernanza del aire para hacer exigibilidad de los planes territoriales de descontaminación del aire. Asimismo, deben fomentarse las tecnologías respetuosas del medio ambiente y la eficiencia energética, e integrar la gestión de la calidad del aire en la planificación urbana y las estrategias de adaptación al cambio climático.

Estas medidas contribuirán a crear un medio ambiente más sano y sostenible para las generaciones presentes y futuras.
